# Role of Injured Pancreatic Extract Promotes Bone Marrow-Derived Mesenchymal Stem Cells Efficiently Differentiate into Insulin-Producing Cells

**DOI:** 10.1371/journal.pone.0076056

**Published:** 2013-09-18

**Authors:** Hongbin Xie, Yunshuai Wang, Hui Zhang, Hui Qi, Hanxin Zhou, Fu-Rong Li

**Affiliations:** 1 The Key Laboratory of stem cell and cellular therapy, the Second Clinical Medical College (Shenzhen People’s Hospital), Jinan University, Shenzhen, China; 2 Laboratory of Cancer Cell Proteomics, Nevada Cancer Institute, Las Vegas, Nevada, United States of America; 3 Department of General Surgery, First Hospital (Shenzhen second People’s Hospital) of Shenzhen University, Shenzhen, China; 4 Shenzhen Institute of Gerontology, Shenzhen, China; National Cancer Institute, United States of America

## Abstract

Mesenchymal stem cells (MSCs) can be successfully induced to differentiate into insulin-producing cells （IPCs) by a variety of small molecules and cytokines *in vitro*. However, problems remain, such as low transdifferentiation efficiency and poor maturity of trans-differentiated cells. The damaged pancreatic cells secreted a large amount of soluble proteins, which were able to promote pancreative islet regeneration and MSCs differentiation. In this study, we utilized the rat injured pancreatic tissue extract to modulate rat bone marrow-derived MSCs differentiation into IPCs by the traditional two-step induction. Our results showed that injured pancreatic tissue extract could effectively promote the trans-differentiation efficiency and maturity of IPCs by the traditional induction. Moreover, IPCs were able to release more insulin in a glucose-dependent manner and ameliorate better the diabetic conditions of streptozotocin (STZ)-treated rats. Our study provides a new strategy to induce an efficient and directional differentiation of MSCs into IPCs.

## Introduction

Mesenchymal stem cells (MSCs) are derived from the mesoderm and have multiple differentiation potentials. Once allogeneically or xenogeneically transplanted into hosts, MSCs can differentiate into cells with mesoderm, ectoderm or endoderm characteristics and contribute to the composition of host tissues by various tissue-specific cues such as micro-environment cytokines, multi-dimensional differentiation signals, extracellular matrix and homogenetic or heterogeneous cell contact [1]. A number of studies have reported that MSCs derived from various tissues can successfully differentiate into functional insulin-producing cells (IPCs) [2-4]. Currently, there are two schemes commonly used to induce adult stem cells to differentiate into IPCs *in vitro*. One is to introduce transcription regulatory factors during the development of pancreatic β-cells by genetic engineering to modulate gene expression that causes the differentiation of stem cells into β-cells or IPCs [5-7]. The other is to supplement specific soluble inducers or small molecule compounds in cell culture media to induce stem cells differentiation into IPCs under conditions that promote β-cells proliferation or differentiation [8-11].

However, current protocols can only induce about 10-20% of stem cells to differentiate into IPCs under *in vitro* induction condition [12]. The resulting IPCs also appear to be not fully maturated, as these cells can usually only secret low levels of insulin which account for about one tenth of normal islet. The efficiency and extent of differentiation of stem cells is modulated by both its internal genetic program and external micro-environment. Evidence suggests that the microenvironment plays an important role in the survival and differentiation of stem cells. How to simulate a microenvironment that mimics the pancreatic developmental stages and cellular differentiation is criitical to successfully manipulate the differentiation of bone marrow-derived MSCs (BMMSCs) into IPCs. Choi et a. have previously found that conditioned medium prepared with the regenerated pancreatic tissues after partial pancreatectomy could induce rat BMMSCs differentiate into IPCs [13], and co-culture of the conditioned media with either isolated adult islets, dispersed islet cells, or mouse β-cell lines was shown to promote pancreatic stem cells differentiation of mature β-cells [14]. Previous studies from our research group have demonstrated that MSCs co-cultured with rabbit pancreatic tissue could greatly stimulate these stem cells to differentiate into IPCs [15]. These findings indicate that the developing and proliferating pancreatic tissues contained some important inducers that are able to promote stem cells differentiate to β-cells. These trans-differentiation factors and soluble proteins secreted by pancreas, therefore, may offer new strategies to efficiently induce stem cells for directional differentiation into IPCs.

In this study, we found that pancreatic tissue extract prepared from the i**njured** rat pancreatic tissue after 60% pancreatectomy can effectively improve the efficiency and maturity of transdifferentiation of BMMSCs into IPCs, thus providing a new strategy for developing an efficient and oriented induction method for inducing stem cells differentiation into IPCs.

## Materials and Methods

### Animals

Sprague-Dawley (SD) rats were purchased from the Experimental Animal Center of Guangdong Province and housed in specific pathogen free rooms of Animal Center of the Second Clinical Medical College, Jinan University. All animal experiments were approved by the Second Clinical Medical College ethics committee, Jinan University and conducted in accordance with institutional guidelines for animal care and use.

### Isolation, culture, and identification of BMMSCs

BMMSCs were collected from the thighbones and shinbones of 4 weeks SD rats. BMMSCs isolation and purification were performed as described by Peister et al [16]. BMMSCs surface markers CD29, CD31, CD34, CD45, CD90, and CD106 (BD Pharmingen, San Diego, USA) were measured by flow cytometry (ALTRA; Beckman Coulter, Inc, Brea, Calif), and their identification was confirmed by adipogenic and osteogenic induction tests as previously described [17].

### Preparation of injured pancreatic tissue extract

Forty SD rats were anesthetized and laparotomy was performed to remove 60% of the pancreas under sterile conditions. The remnants of pancreatic tissue were harvested at 72 hours after the abdomen was closed. Pancreatic tissue extract was prepared as previously described by Hardikar et al. [18]. Pancreatic tissues were collected after partial pancreatectomy, rinsed with cold 1×PBS, and stored in PBS solution (10 µL: 1 mL) containing protease inhibitor (630 U/mg; Roche, Basel, Switzerland) at 4°C. Pancreatic samples were homogenized with a tissue homogenizer (Polytronpt-DA2105/2EC, Kinematica, Switzerland) and centrifuged at 3000 rpm for 10 minutes at 4°C. The supernatant was further centrifuged at 1,2000 rpm for 20 minutes at 4°C and filtered with 0.22-µm filtration membrane (Millipore, USA). The protein concentration of the extract was determined between 3.54^~^3.86 mg/mL by the BCA kit (Pierce, Rockford, USA). The prepared pancreatic extracts were frozen at -80°C for further use.

### Separation and purification of rat islets

SD rat islets were isolated and purified as described previously by our laboratory [19]. The islets were collected with RPMI-1640 culture medium containing 20% fetal calf serum after islet isolation and purification.

### BMMSCs differentiate into IPCs

BMMSCs at passage 3 were used for induction to IPCs. For this purpose, cells with 80% confluency were removed from the 25-cm^2^ flasks by trypsin and seeded into six-well plates at the density of 2-3×10^5^/well. Cells were cultured in 10% fetal bovine serum high glucose DMEM(25 mmol/L). The medium was not changed for the first five days. When cells reached 80% confluency, they were divided into four groups, each group treated with differently induction method, they were induced to differentiate into iPCs. Traditional group: cells were treated with a two-stage protocol [20]. Stage 1: the cells were added with 3mL induction solution 1 (DMDM/F12 culture medium; containing 20µg/L bFGF, 10 µg/L EGF and 2% B27) and cells were cultured for 7 days. Stage2: the cells were washed three times with PBS and were added with 3 mL induction solution 2 (serum-free HG-DMEM culture medium; containing 10 µg/L betacellulin(BTC), 10 µg/L HGF, 10 µg/L activin-A, 10 mmoL/L nicotinamide and 2% B27) and cells were cultured for additional 7 days. Pancreatic extract group: Each well was added with injured pancreatic tissue extract (400 mg proteins/L) and 3 mL LG-DMEM culture medium containing 10% fetal bovine serum, cells were cultured for 14 days. The medium was changed every 3 days. Mixed group: cells were cultured for 14 days with injured pancreatic tissue extract at 400 mg proteins/L, based on the traditional two-stage protocol. The culture medium was changed every 3 days. Control group: the cells were only added with 3 mL LG-DMEM culture medium containing 10% fetal bovine serum, with the medium replenished every 3 days.

### Morphology and dithizone staining

During this time, the morphological changes were observed by phase contrast microscopy every day (Olympus CK40, Japan). DTZ (Sigma) stock solution was prepared as previously reported [19] by dissolving 100mg of DTZ in 5 ml of DMSO. The cells induced for 14 days were washed with 1×PBS buffer (pH 7.4) twice and added with 1 mL 1×PBS buffer and 10 µL dithizone stock solution, storing at 37 °C incubator for 15 minutes. The crimson-red- stained clusters were examined with a phase-contrast microscope.

### Immunofluorescence detection

The IPCs were collected, fixed with 4% paraformaldehyde for 10 minutes, rinsed with 1×PBS, and treated with 0.3% Triton X-100 for 10 minutes. After blocking with 5% bovine serum albumin at 37 °C for 1 hour, the IPCs were incubated with rabbit anti-rat Pdx1 antibody, rabbit anti-rat Insulin antibody and goat anti-rat C-peptide antibody at 37 °C for 1 hour. Cells were further incubated with FITC-labeled goat anti-rabbit polyclonal antibody (Jackson ImmunoResearch) and PE-labeled donkey anti-goat (Rockland) at 37 °C for 1 hour. Cells were counterstained with DAPI at 10 µg/mL, at room temperature for 8 minutes, and washed with 1×PBS three times. Cells were mounted with anti-quench reagent and observed under fluorescent microscope (ECLIPSE TE2000-U, Nike, Germany).

### RT-PCR and Quantitative Real-Time PCR(qRT-PCR)

The IPCs were collected and total RNA was extracted using the Trizol kit (GIBCO, USA). The concentration and purity of the isolated RNAs were determined with a UV spectrophotometer (Eppendorf, USA) and the RNA quality was examined in 1% agarose gel electrophoresis. Reverse transcription reaction was conducted in accordance with the instructions of the RevertAid^TM^ First Strand cDNA Synthesis Kit (MBI, Canada). PCR reaction was performed in accordance with the Platinum PCR program, the reaction system was 50 µL and cDNA was 2 µL. β-actin RNA was used as an internal reference. 5µL PCR products underwent 1.5% of common agarose gel electrophoresis and image was monitored with the gel electrophoresis imaging system. The primers used are shown in Table 1.

**Table 1:** List of primer and condition information for RT-PCR.

gene	primer sequence(5’–3’)	annealing temperature	cycle number	size of segment(bp)
Pdx1	GGTGCCAGAGTTCAGTGCTAATCC	58°C	35	113
	GACTTCCCTGTTCCAGCGTTCC		
Isl1	CACAAACAGCCCGAGAAGACC	58°C	35	167
	TGAAACCAGACCCGGATGACT		
Glut2	CCAGCACATACGACACCAGACGC	58°C	35	136
	CAAGCCACCCACCAAAGAACGAG		
Nkx6.1	CCGCCAAGAAGAAGCAGGAC	54°C	35	154
	CTGCCTCCGCTGGATTTGT		
PC1	TGGTGAATGTTGTGGAGGAGAA	54°C	35	155
	AGCACTTTGTAGGAGCCGTAGC		
PC2	TGCTCACCTCCAAGCGAAACCAG	54°C	35	106
	GCATCAAGGACTCCGTAGCCAAA		
Ngn3	CACTGAGCAAGCAGCGACGAA	58°C	35	152
	GCGCAGGGTCTCGATCTTTGTA		
SSt	CAGGAACTGGCCAAGTAC A	54°C	35	120
	GTTCTTGCAGCCAGCTTTG		
β-actin	CCCATCTATGAGGGTTACGC	54°C	35	150
	TTTAATGTCACGCACGATTTC		

The qRT-PCR was performed with Quantitect Sybr, Green PCR Kit (Qiagen, Germany) using the Roche LightCycler 2.0 (Roche Diagnostic, Germany). Reactions were performed in triplicates. Specificity of the amplified products was determined by melting peak analysis. Quantification for each gene of interest was performed in relation to a standard curve represented by the appropriate cDNA plasmid. Quantified values were normalized against the housekeeping gene β-actin. Each experiment for the three times.

### Electron microscopy

The IPCs were fixed in 0.1% glutaraldehyde/2% formaldehyde in 0.1M cacodylate buffer, transferred to 0.1M cacodylate buffer, and embedded in Epon812 and cut with Ultracut UC Tultra microtome(Leica, Bensheim, Germany). Specimens were then stained with acetic acid U and lead citrate fluid sequentially and viewed by electron microscopy (Philips, Amsterdam, Holland) operated at 80kV.

### Differentiation efficiency detection

The IPCs were collected and adjusted to 1 × 10^6^ cells/mL 100 µL cell suspension was fixed with 4% paraformaldehyde (Sigma, St. Louis, MO) for 15 min. They were permeabilized with 0.1% Trion-X 100 (Sigma) and blocked with 2.5% fetal calf serum. The cells were added with rabbit anti-rat C-peptide antibody (Santa Cruz, California, USA). The cells were incubated at 4 °C for 30 minutes, The cells were washed three times with 1×PBS, and incubated with FITC-labeled goat anti-rabbit antibody (Rockland, PA, USA) at room temperature for additional 30 minutes. Rat IgG1-FITC (Ancell,USA ) was used as an isotype control. The cells were analyzed by flow cytometry.

### Glucose stimulation experiment

The IPCs in each group were collected, rinsed with 1×PBS, and adjusted to 1 × 10^5^ cells/mL. 1 mL cell suspension was incubated in each of 24-well culture plates and incubated with DMEM/F12 culture 1 mL containing 5.5 mmol/L glucose and 25.5 nmol/L glucose for 1 hour. The culture medium was collected. The level of insulin released into the culture media was determined using the rat ELISA kit (Mercodia, USA). Fifty islets at islet equivalent(IEQ) = 150 µm were selected, taking as a control group.

### Establishment of diabetes mellitus models and cell transplantation

To set up models of rats with diabetes, A total of 24 male, 4- to 6-weeks- adult SD rats, weigh between 200-250g were intraperitoneally injected with streptozotocin (STZ; 30/kg; Sigma, USA). Diabetes was monitored by measurement of blood glucose concentration from the tail vein using a blood glucose device (Roche Accu Check III, Roche Diagnostics, Basel, Switzerland). Rats with non-fasting blood glucose 300g/L for 3 consecutive days were considered as onset of diabetes. Seven days after STZ injection, diabetic rats were used for transplantation. After anesthetization, diabetic rats were randomly divided into 4 groups: Diabetes group(n=6): a sham transplant of 0.1ml PBS were transplanted under the right kidney capsule. Traditional group(n=6), Pancreatic extract group(n=6) and Mixed group(n=6): 1×10^7^ differentiated IPCs suspended in 0.1 ml PBS from differently group were transplanted under the right kidney capsule. Six normal male SD rats as Normal group underwent the same procedure, but were only injected with 0.1ml PBS. Fasting blood-glucose levels were tested 2 days after transplantation and then monitored with accutrend strips(Roche Diagnostics, Basel, Switzerland) every 5 days after transplantation for 30days.

### Statistical processing

Data were expressed as mean ± SD and analyzed with SPSS 10.0 software. Mean values between the two groups were compared using independent samples *t*-test. A level of *P* < 0.05 was considered statistically significant difference.

## Results

### Isolation, culture and identification of BMMSCs

**Figure 1:**
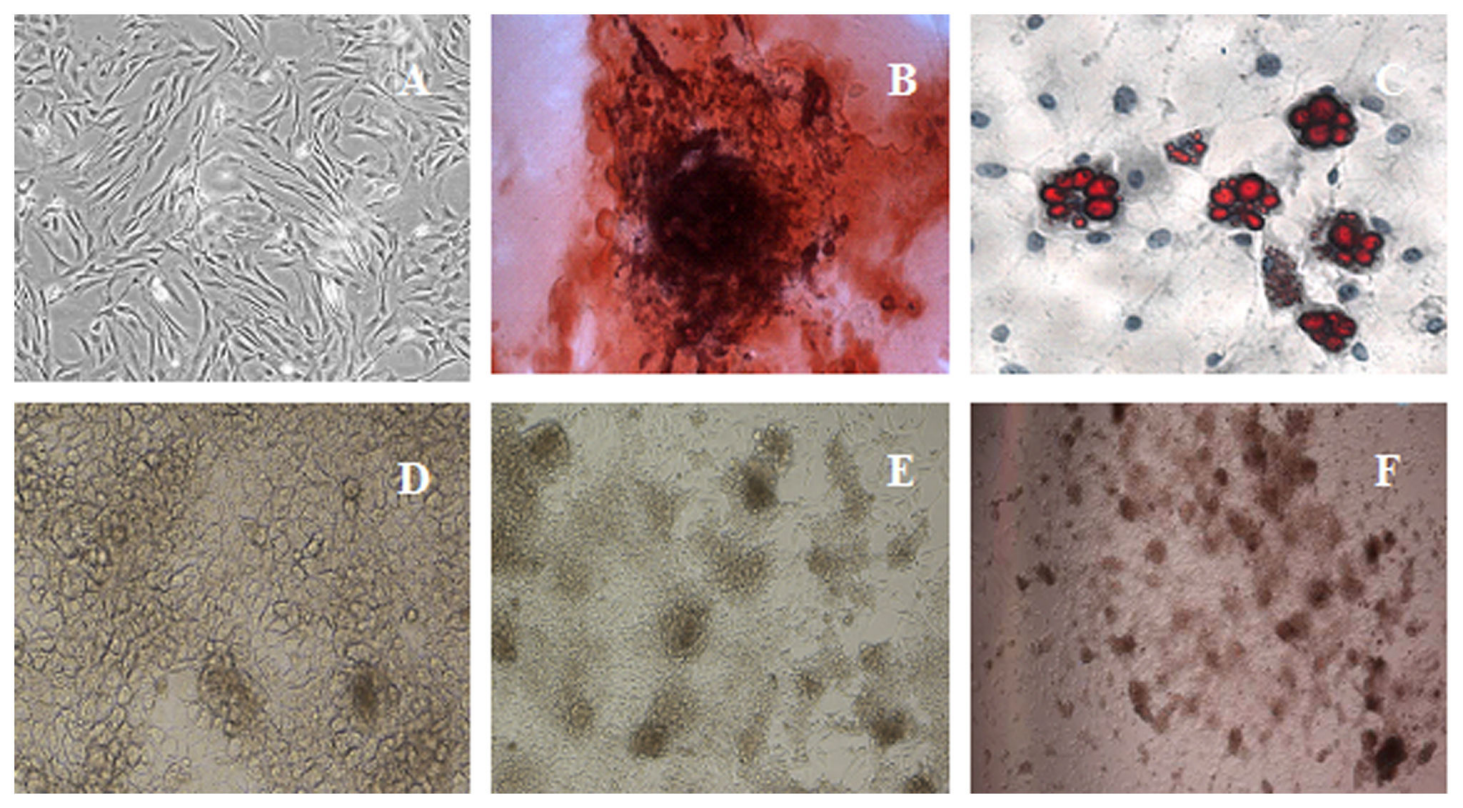
Identification of BMMSCs differentiated into IPCs. A. Purifiation of at passage 3 BMMSCs(×200 magnification); B.The osteogenic phenotype was revealed by staining with Alizarin red, which stains calcium-rich mineral deposits (×200 magnification). C.For the adipogenic cells, intracellular accumulations of lipid droplets were stained with Oil red O (×200 magnification). D.BMMSCs difference from mixed group following 7 days (×200 magnification); E. IPCs from mixed group were formed in 14 days(×100 magnification); F. IPCs from mixed group were stained with DTZ(×40 magnification).

The isolated and purified BMMSCs were shaped as elongated spindle, showing a vortex-like growth (Figure 1A). Surface markers of the cells at passage 3 detected by FACS showed they were positive for CD29 (98.8%), CD90 (98.4%) and CD106 (92.2%), while negative for CD45 (3.0%) and CD31 (2.6%). We also evaluated the osteogenic and adipogenic differentiation potential of expanded BMMSCs. BMMSCs cultured in osteogenic medium formed mineral deposits exhibited as von Kossa positive (Figure 1B). When dispensed in adipogenic medium for 21 days, Oil-Red O-positive adipogenic cells were observed (Figure 1C).

### Morphology and dithizone staining during the differentiation

BMMSCs at passages 3-5 were treated with differently protocol and their morphologic characteristics were detected every day. Undifferentiated BMMSCs were of typical spindle-like shape. In three induction groups, during 3 days of induction, cells turned round(Figure 1D), during 7 days of induction, accumulated together and like cobble stones (Figure 1E). The cells aggregated in clusters and islet-like cell clusters were formed at 14 days (Figure 1F). Mixed group: Cells aggregate were markedly increased and their boundary became clearer at 14 days. Derived clusters were proved to be DTZ positive (Figure 1G), while undifferentiated cells were negative. DTZ can specifically stain insulin granules in β cells.

## RT-PCR and qRT-PCR

**Figure 2:**
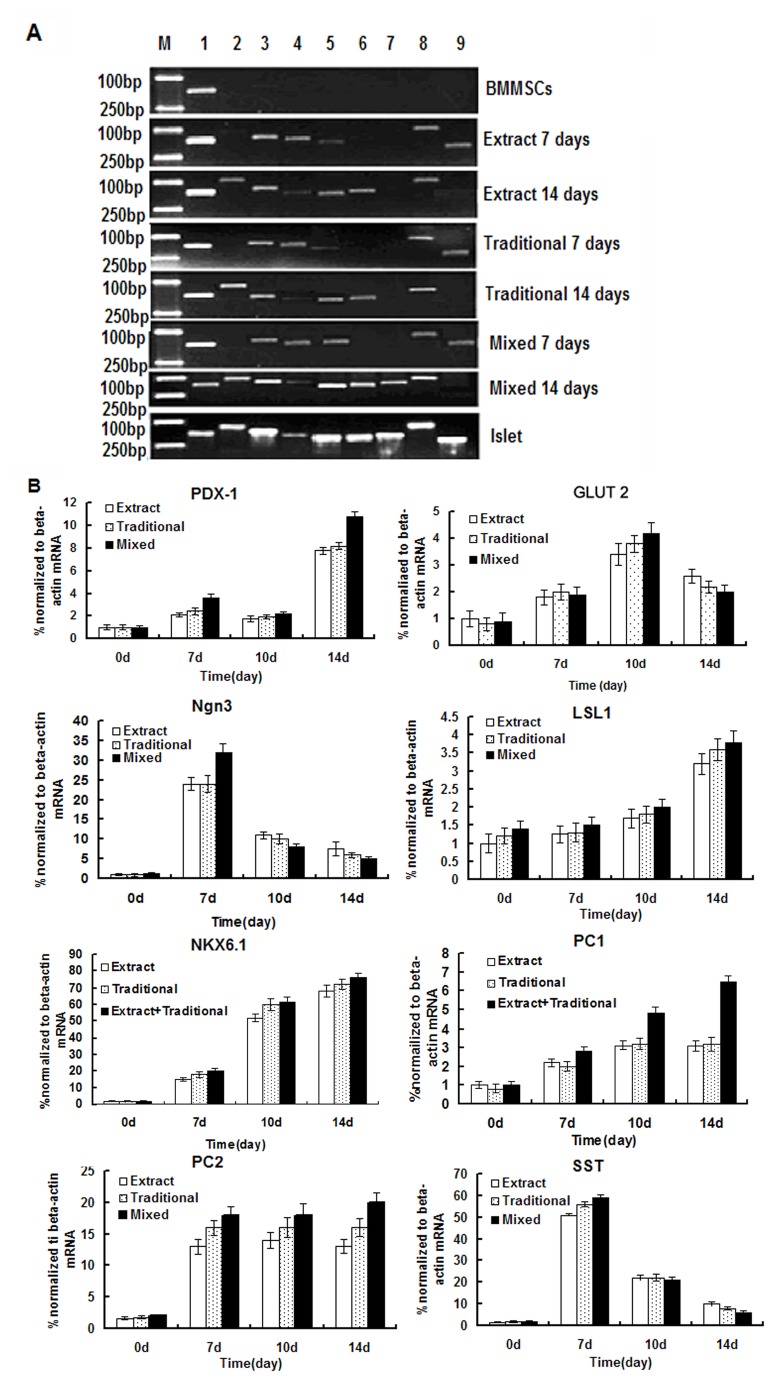
RT-PCR and Quantitative real-time PCR analyses for the expressions of pancreatic β-cell development-related and insulin production related genes. A. day 7 and 14 differentiated IPCs were analysed RT-PCR (M: marker; l:β-actin, 2:PDX-1, 3:g1ut2, 4:Ngn3, 5:Isl1, 6:Nkx6.1, 7:PC1, 8:PC2, 9:Sst); B. Quantitative real-time PCR analyses for the expressions of PDX-1, g1ut2, Ngn3, Isl1,Nkx6.1,PC1, PC2 and Sst. Results are the means ± SEMs for 3 times.

The expression of genes related to islets in derived IPCs was assessed by RT-PCR(Figure 2A). No expression of PDX-1, g1ut2, Ngn3, Isl1, Nkx6.1, PC1, PC2 and Sst was detected in undifferentiated BMMSCs. In three induction group, the g1ut2, Ngn3, Isl1, Nkx6.1, PC2, Sst genes became detectable in RT-PCR at 7 days, but PDX-1 was still weakly expressed. PDX-1, g1ut2, Ngn3, Isl1, Nkx6.1, PC2 and Sst showed a sustained expression in Traditional group and Pancreatic extract group at 14 days, and the expression of all the above genes in Mixed group was further enhanced greatly than that in traditional group and pancreatic extract group.

Quantitative real time PCR showed (Figure 2B) that Ngn3, Sst and PC1 mRNA were significantly increased in 7days, Glut2, NKX6.1 and PC2 mRNA were significantly increased on 10 days, PDX1, lsl1, Nkx6.1 and PC1 mRNAs were significantly increased on 14 days in three induction group. The expression of Glut2, lsl1, PDX1, Nkx6.1, PC1 and PC2 genes were gradually up-regulated during the induction stage. The gene expression of mixed group was extremely enhanced than that in traditional group and pancreatic extract group. The expression of Ngn3 and SST was up-regulated in 7days and was down-regulated in 10 and 14 days. Thus BMMSCs had the capability to differentiate into β-Cells and express genes associated with insulin production in our analysis.

### Immunofluorescence staining

**Figure 3:**
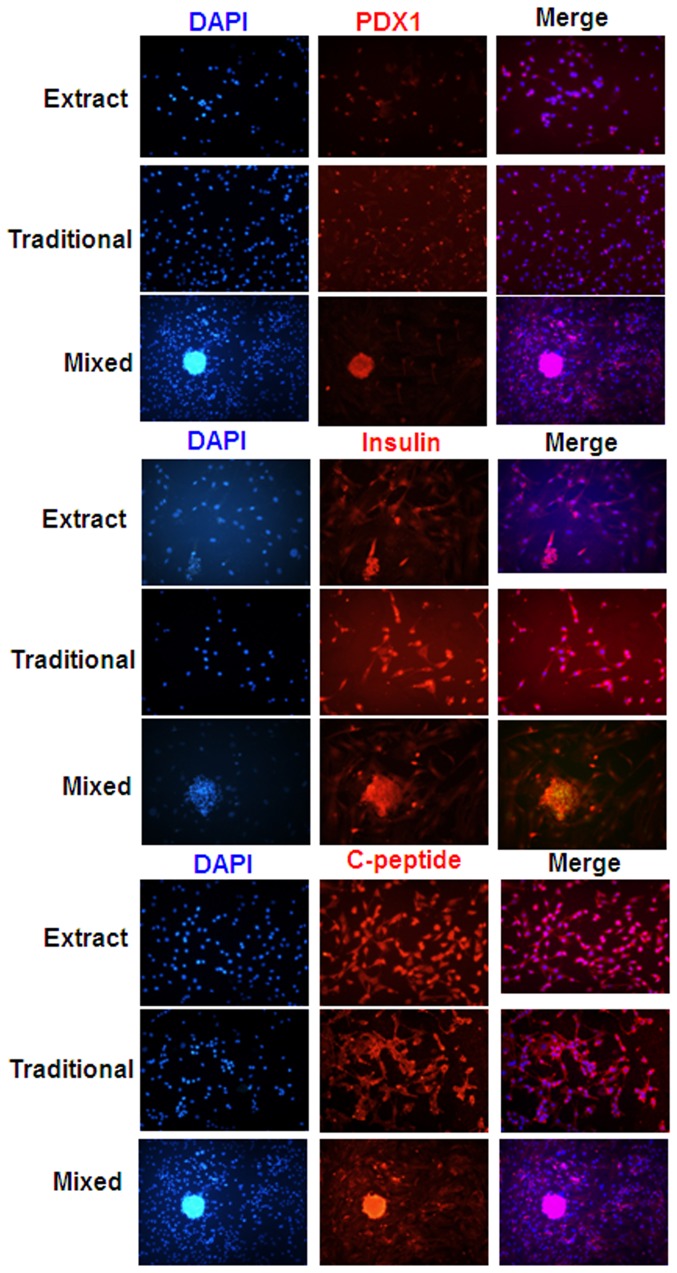
Immunofluorescence reveals BMMSCs differentiated into IPCs in vitro. The IPCs at days 14 were fixed and stained with antibodies against PAX-1, insulin and C-Peptide(red) and visualized with secondary antibody. 4′,6-diamidino-2-phenylindole (DAPI) was used to counter-stain DNA (blue). Microphotographs were taken with a fluorescence microscope. Scale bar: 100 µm.

The expression of endocrine hormones like PDX1 (red), insulin (red) and C-peptide (green) in derived IPCs was investigated by immunofluorescence. Undifferentiated BM-MSCs counterstained with PDX1, insulin and c-peptide primary antibodies served as negative controls. The IPCs were found to have positive expression of these genes. The level of positive expression of these genes in mixed group was significantly higher than that traditional group and pancreatic extract group (Figure 3). All were counterstained by using DAPI for nuclear staining.

### Differentiation efficiency

**Figure 4:**
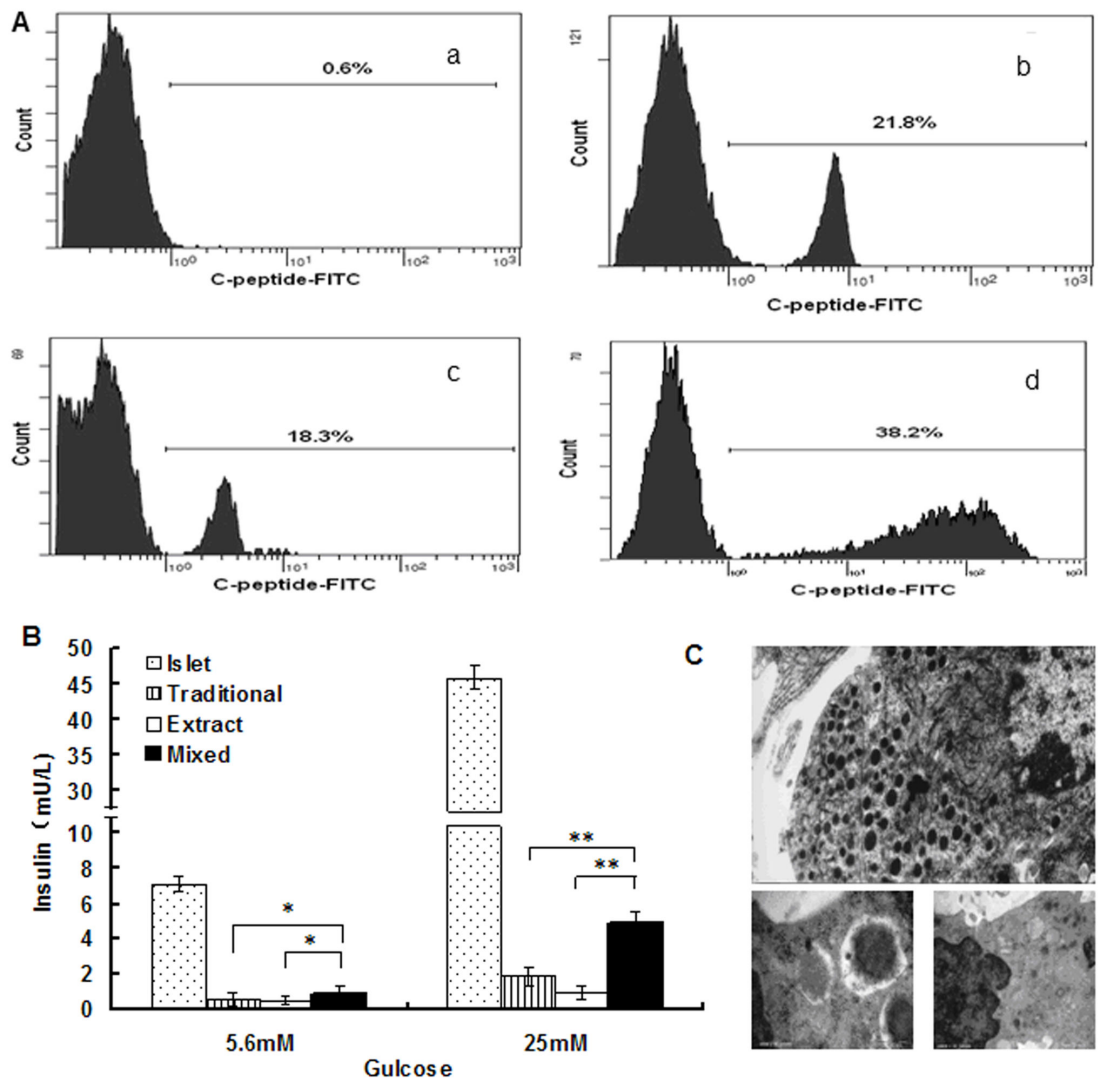
Physiological functions of IPCs derived from BMMSCs. A. The expression of C-peptide from differentiated IPCs was analyzed with FCM. (a. BMMSCs were cultured in the growth medium as negative controls. b. The expression of C-peptide from traditional group was analyzed. c.The expression of IPCs from pancreatic extract group was analyzed. d. The expression of IPCs from Mixed group was analyzed. The respective immunoglobulin isotypes were used as isotype controls). B. Differentiated IPCs release insulin in response to glucose. Glucose-induced insulin release data correspond to the amount of insulin secreted for 1 h after 5.5mM and 25mM glucose stimulation. Data presented are means ± SD of the triplicate wells of the same IPCs culture. C. IPCs showed secretary vesicles with a dense core and a peripheral, electron-lucent halo, and mitotic activity was detected by conventional electron microscopy (Scale bar: 500 nm).

The expression of C-peptide from differentiated IPCs was analyzed with FCM. We found that the positive expression of C-peptide was 0.8 ± 0.3% in negative controls, 21.8 ± 2.2% in the Traditional group, 18.3 ± 1.8% in Pancreatic extract group, and 38.2 +3.4% in Mixed group, respectively (Figure 4). The labeling index in mixed group was significantly higher than that in Traditional group and Pancreatic extract group (*P* < 0.05; Figure 4A).

### Insulin secretion assay

In the glucose challenge test, undifferentiated BMMSCs showed no obvious release of insulin. Under the stimulation of high concentrations of glucose (25mM), the levels of secreted insulin was 4-6 folds higher than that of cells under the stimulation of low concentration of glucose (5.6 mM). Compared with natural islet, the insulin release in IPCs in Traditional group was only 1/30 and one thirty-fifth of natural islet, that in Pancreas extract group was 1/40 and one forty-seventh of natural islet, and that in Mixed group was 1/8 and one-tenth of natural islet. The Mixed group was significantly higher than Traditional group and Pancreatic extract group (*P* < 0.05; Figure 4B).

### Ultrastructural characteristic of IPCs

Differentiated IPCs from differently induce group showed atypical structure of secretory cells with abundant endoplasmic reticulum, mitochondria and secretory vesicle sunder electron microscopy examination(Figure 4C).

### Function tests of IPCs in vivo

**Figure 5:**
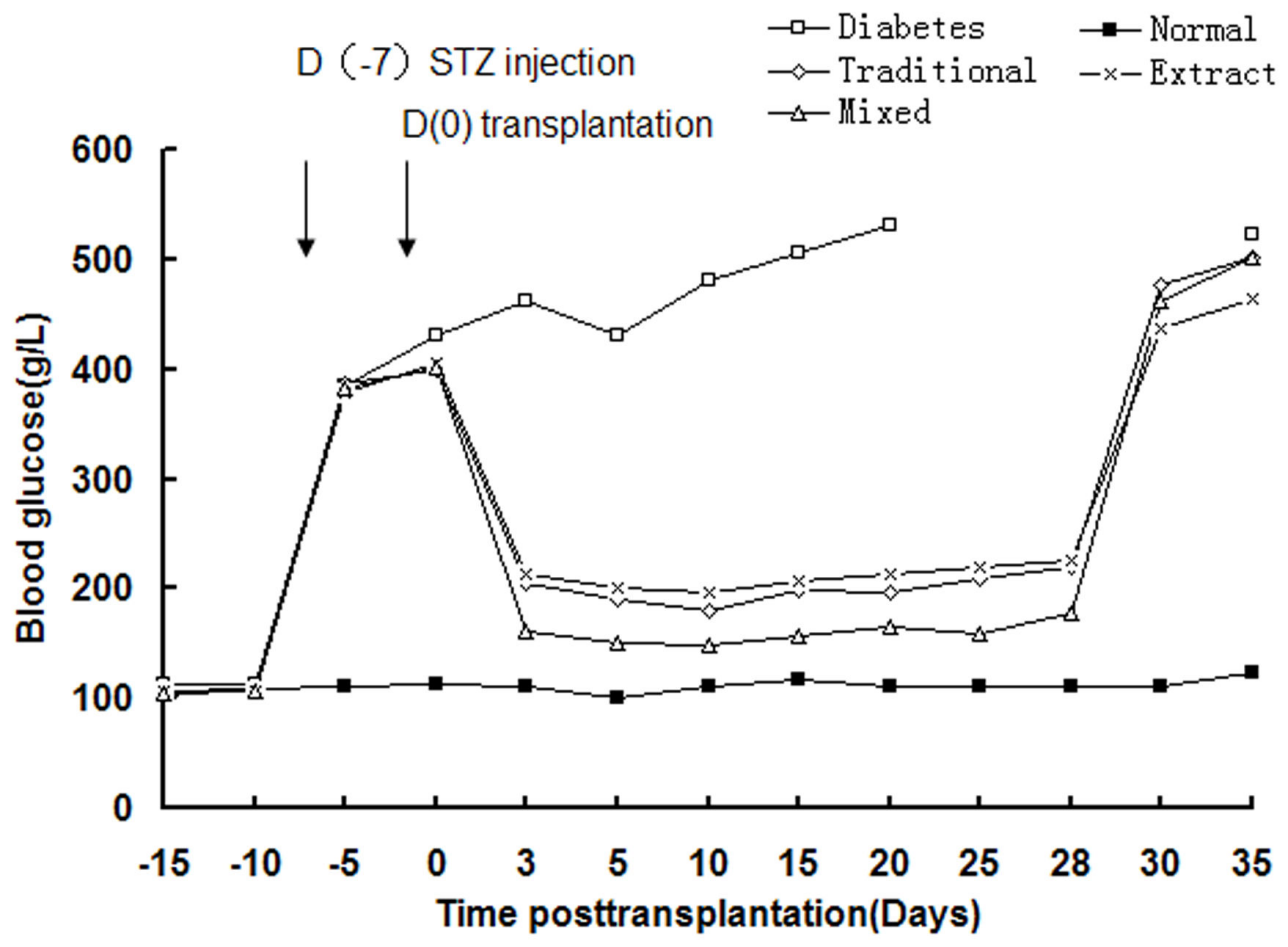
Transplantation of differentiated IPCs into STZ-treated diabetic rats normalizes blood glucose levels. Glucose levels were monitored every 5 days after transplantation. Diabetes: glucose levels in sham PBS implanted in diabetes rats (n=6); Traditional: glucose levels in BMMSC-derived IPCs implanted in diabetes rats that were induced with the traditional two-stage protocol (n=6). Extract: glucose levels in BMMSC-derived IPCs implanted in diabetes rats that was induced with injured pancreatic tissue extract (n=6). Mixed: glucose levels in BMMSC-derived IPCs implanted in diabetes rats that induced with injured pancreatic tissue extract, based on the traditional two-stage protocol (n=6). Normal: glucose levels in normal rats (n=6). The left kidney was removed at 28 days after transplantation. Data presented are means ± SD.

The potential of derived IPCs from three different induction groups to normalize hyperglycemia in vivo was investigated in STZ- induced diabetic rats. a sham transplant of PBS (n=6) or derived IPCs from three different induction groups (n=6) were transplanted under the right kidney capsule. With in 2–3days, blood glucose levels in three differentiated IPCs transplantation model began to decrease and got close to normal in the period observed. In those study groups, the blood glucose level of Traditional group and Pancreas extract group were gradually reduced on 3 day after transplantation, to maintain at around 200g/L from 3 days to 28 days after transplantation. The blood glucose in mixed group was 150g/L on day 3 after IPCs transplantation. The blood glucose level of mixed group was significantly differently than that in the traditional group and the pancreatic extract group (*P* < 0.05; Figure 5). Diabetic group sham transplant of PBS, persistent hyperglycemia was observed and all rat died within 30 days after transplantation. In addition, after removal of the left kidney transplanted with derived IPCs from three different induction groups on day 28, the blood glucose level of diabetic rats reversed to greater than 300g/L within 2 days. These results showed that BMMSC-derived IPCs, when transplanted into the renal capsule, were functional *in vivo* and capable of reversing hyperglycemia in diabetic rats.

## Discussion

MSCs have been considered the ideal stem cell candidate for induced differentiation of islet cells, because of multiple differentiation potentials [21,22], easy to sample and no immune rejection. Many scholars are trying to find an effective method for inducing MSCs differentiate into IPCs *in vitro*. A number of studies have shown that MSCs derived from different tissues can be induced to some extent to express markers related to pancreatic endocrine cells and to secrete insulin, however, the efficiency of differentiation was very low [23-25]. In this study, the traditional two-step induction method was utilized, in brief, BMMSCs were induced with DMDM/F12 medium containing bFGF, EGF, and B27 into nestin-positive cells, and nerve stem cells were amplified with bFGF and EGF as a mitogen combination [26]. Then, activin-A, β cell regulin, hepatocyte growth factor, nicotinamide and other cytokines were supplemented with serum-free high-glucose medium (4.5 mmol/L). As the glucose concentration is crucial in the induction and differentiation process, 4-5 mmol/L glucose can increase insulin synthesis, while 20-30 mmol/L could inhibit β-cell differentiation. Nicotinamide can promote fetal pancreatic cell differentiation and increase β-cell quantity, also is conducive to the synthesis of insulin. In addition, activin-A and BTC can promote MSCs differentiation into β-cell.

The present study demonstrated that BMMSCs were able to adopt a pancreatic endocrine phenotype morphologically and functionally in vitro under a defined two-stage induction protocol without any genetic modification. Furthermore, these derived IPCs could secrete insulin in a glucose-dependent manner in vitro, and could alleviate the hyperglycemia of STZ-induced diabetic rats when transplanted under the renal capsule. Despite the low insulin production in vitro, these results indicate that BMMSCs may be an available candidate to alleviate limitations of islet cell replacement.

Despite of the various protocols have been used to induce the differentiation of rodent BMMSCs to IPCs in vitro, the best conditions and the detailed mechanism required for the differentiation have not been entirely understood. Hardikar et al. have removed 80-90% pancreas in STZ-induced diabetic mice and found that the blood glucose of mice still maintained within the normal range after surgery, indicating that the residual pancreas has a strong regenerative capacity although the diabetes existed, they can secrete cytokines to promote regeneration of pancreatic β-cells [27]. Then the pancreas extract solution remained in mice was intraperitoneally injected into the diabetic mice and found to reduce blood glucose levels for 190 days [28]. Kanitkar et al. [29] obtained the similar results after rat pancreatic cell culture supernatant was intraperitoneally injected to diabetic rats, indicating that the proliferating pancreas can secrete a large amount of cytokines to promote regeneration of pancreatic β-cells. The injured pancreatic tissue extract promote BMMSCs differentiate into islet-like cells, was shown to significantly increase the IPCs capacity of secreting insulin [30]. The above studies have confirmed that during the pancreatic repair or development process, under certain microenvironments, the damaged or developing pancreatic tissues are doomed to secrete large amounts of pancreatic development-related cytokines and transcription proteins [31], thus promote regeneration of pancreatic β-cells or stem cells in the pancreas differentiate into pancreatic β-cells. This finding provides a new idea for stem cell differentiation to IPCs.

In this study, we report that the BMMSCs were co-cultured with injured pancreatic tissue extract and the traditional two-stage protocol. Our results demonstrate that injured pancreatic tissue extract could effectively promote the trans-differentiation efficiency and maturity of IPCs by the traditional induction. Moreover, IPCs were able to release more insulin in a glucose-dependent manner and ameliorate better the diabetic conditions of streptozotocin (STZ)-treated rats. In our primary study, BMMSCs were induced with a traditional two-stage protocol. EGF,bFGF,HGF,BTC,activin-A and high glucose combined with nicotinamide could not effectively induce the formation of IPCs. C-peptide positive rate was 21.8% in IPCs. Under the stimulation of low- and high-concentrations glucose, the capacity of differentiated IPCs releasing insulin was 1/31 and one thirty-fifth of natural islet. The conclusion was consistent with the majority of IPCs after differentiation in vitro [32]. This is evidence that the obtained IPCs were not too mature to produce enough insulin. It may be associated with PI transformation disorder [33,34]. PC1 and PC2 is two key enzymes contributing to promote pro-insulin transform into insulin, mainly distributes in the pancreatic β-cells, their normal expression is the key restriction enzyme leading to pro-insulin transformation and metabolism into insulin, while the deficiency may hinder the transformation into insulin, nor express the mature biologically active insulin [35,36], thus cannot secrete insulin. PC1 was not expressed in the RT-PCR, indicating that the induced IPCs here had appeared pro-insulin transformation disorder. This is also the reason for low trans-differentiation efficiency and poor maturity of traditional induction methods.

Rat pancreatic extract was reported to be a potent inducer of pancreatic islet differentiation [37]. Damaged pancreas extract contains some specific β cell regeneration and stem cell differentiation factors, They are able to promote stem cell differentiation, β cell proliferation and the secretion of insulin [38]. We carried out proteomic analysis of proteins from injured rats pancreatic extracts, some proteins were found to be associated with pancreatic development and differentiation. These proteins may provide new transcription factors for the efficient and directed differentiation of stem cells into IPCs. In addition, micro-environment *in vivo* governs stem cell differentiation and plays a pivotal role in cell fate determination. Studies have shown that the induced differentiation of stem cells is controlled by their genetic program and micro-environment, which determines the fate of adult stem cells differentiation [39,40]. Therefore, an efficient *in vitro* induction program must meet two conditions: (1) the gene expression and signal control for normal development of pancreatic β-cell; (2) the micro-environment for normal development of pancreatic β-cells. These two factors are the key for BMMSCs differentiation into IPCs in vitro. In this study, we combined traditional induction method that regulated pancreatic development-related genes with the pancreatic micro-environment approaches, found that the Mixed group could induce more IPCs with large sizes at 14 days after the induction, compared with the traditional group and pancreatic extract group, the dithizone staining was more apparently red. This is evidence that cell group contained more zinc ions, which acted with more zinc ion chelating agent. At 7 days after the differentiation, the expression of β-cell development-related gene g1ut2, Ngn3, Isl1, Nkx6.1, PC2, Sst was observed, while the β-cell development-related PDX-1, g1ut2, Ngn3, Isl1, Nkx6.1, PC1, PC2, Sst were found to sustained expression at 14 days. The results indicated that the differentiated IPCs have the biological characteristics of islet β-cells, in which the expressions of two key enzymes PC1 and PC2 has confirmed that disorder of pro-insulin transforming to insulin is solved. Immunofluorescence staining showed that the PDX-1, Insulin and C-peptide expression was positive. There were 38.2% of differentiated cells expressing C-peptide, under the low- and high-concentration glucose stimulation, the capacity of induced cells to release insulin was 1/8 and one-tenth of normal islets. The results indicated that a combination of pancreatic tissue extract and traditional inductor is superior to one of them alone in terms of inducing BMMSCs differentiate into IPCs, the efficiency of trans-differentiation and the maturity were significantly improved. The maturity of IPCs after differentiation is close to natural islet. When they were transplanted under the renal capsule, it was amazing that the blood glucose level was significantly decreased on day 3 after transplantation. The blood glucose level of mice with mixed group transplantation was significantly lower than that of Traditional group and Pancreatic extract group. After removal of left kidney on day 28 after transplantation, the blood glucose was markedly elevated. These results showed that derived IPCs were functional *in vivo* and capable of reversing hyperglycemia in diabetic rats.

In conclusion, our study presents the phenomenon that BMMSCs can be induced to transdifferentiate into IPCs both phenotypically and functionally *in vitro* is regulated by its intrinsic genetic program and surrounding micro-environment. Pancreatic tissue extract can effectively improve the trans-differentiation efficiency and maturity of IPCs by the traditional induction. Moreover, the derived IPCs possess the function of insulin release in response to glucose stimulation and ameliorate better the diabetic conditions of streptozotocin (STZ)-treated rats. Our study provides a new strategy to induce an efficient and directional differentiation of MSCs into IPCs.
